# Optimization of Reactive Diluent for Bio-Based Unsaturated Polyester Resin: A Rheological and Thermomechanical Study

**DOI:** 10.3390/polym13162667

**Published:** 2021-08-10

**Authors:** Pavle Spasojevic, Sanja Seslija, Maja Markovic, Olga Pantic, Katarina Antic, Milica Spasojevic

**Affiliations:** 1 Faculty of Technical Sciences, University of Kragujevac, Svetog Save 65, 32000 Cacak, Serbia; pspasojevic@tmf.bg.ac.rs; 2 Institute of Chemistry, Technology and Metallurgy, Center of Excellence in Environmental Chemistry and Engineering, University of Belgrade, Njegoševa 12, 11000 Belgrade, Serbia; 3 Innovation Center of Faculty of Technology and Metallurgy, University of Belgrade, Karnegijeva 4, 11000 Belgrade, Serbia; mmarkovic@tmf.bg.ac.rs (M.M.); katarina.antic@tmf.bg.ac.rs (K.A.); 4 Faculty of Technology and Metallurgy, University of Belgrade, Karnegijeva 4, 11000 Belgrade, Serbia; opantic@rocketmail.com; 5 Innovative Centre of Faculty of Chemistry, University of Belgrade, Studentski Trg 12-16, 11000 Belgrade, Serbia; smilica84@gmail.com

**Keywords:** unsaturated polyester resins, dimethyl itaconate, methyl methacrylate, rheological properties

## Abstract

Nowadays, unsaturated polyester resins (UPR) are mainly obtained from non-renewable resources. The ever-increasing regulations and the continuous demand for more sustainability have led to extensive research towards more environmentally suitable alternatives to petroleum-based materials. However, one of the main disadvantages of bio-based UPR is their relatively high viscosity compared to petrochemical ones. In order to overcome this drawback, in this work, we investigated the possibility to lower the resin viscosity utilizing a mixture of dimethyl itaconate (DMI) and methyl methacrylate (MMA) as a reactive diluent. The effect of the DMI and MMA ratio on resin rheological properties was investigated. The optimal curing parameters were determined and all UPRs had a high gel content, which was shown to be dependent on the DMI and MMA ratio in the formulation. Furthermore, thermomechanical and mechanical properties of the resulting network were also found to be affected by the used reactive diluent mixture. A small substitution of DMI by MMA proved to be advantageous since it offers lower resin viscosity and improved mechanical properties.

## 1. Introduction

Unsaturated polyester resins (UPRs) are one of the most essential classes of polymers and highly versatile as they can easily be tailor-made to achieve the desired properties. Since the middle of the 20th century, when commercial production started, UPRs have been used with great success in many industrial sectors [1]. These polymers have been the center of a real revolution in the boat industry, as they can provide great performances and a very high flexibility of use. Owing to their great design versatility, light weight, easy handling, lower system costs and mechanical strength, UPRs are also commonly used in the automotive sector. UPRs have been applied as building materials, especially in the manufacture of hobs for cookers, tiles for roofs, bathrooms accessories, but also pipes, ducts and tanks [2].

The chemistry of UPRs involves preparation of unsaturated polyesters (UPs) through a polycondensation process, where a dihydroxy compound (or a mixture of dihydroxy compounds) reacts with anhydrides or dicarboxylic acids [3]. The unsaturation of the polymer backbone allows curing of UPs with an unsaturated monomer via radical polymerization reactions. The incorporation of low molecular weight monomers as reactive diluents (RDs) enables fine-tuning of the cured UPR properties, such as the viscosity, processability, thermal and mechanical properties [4]. It is important to note that all reactants involved in the synthesis contribute to the final set of UPR properties. The most commonly used reactants originate from petrochemical sources, and therefore the toxicity and volatility of these materials require careful environmental, health and safety monitoring.

One of the biggest issues related to these formulations arises from the use of styrene as a RD, since styrene is highly volatile and has been identified as a hazardous air pollutant. On the other hand, growing concerns over the greenhouse effect and the unpredictable oscillation of crude oil prices have directed research towards finding an adequate alternative to styrene as one of the most important industrial targets [5].

Therefore, the use of bio-derived platform molecules in polymer synthesis provides twofold benefits: freeing the polymer industry from dependence on crude oil and reducing the environmental impact of polymer synthesis due to the inherent functionality of bio-derived platform molecules. This has encouraged researchers and scientists to hasten the integration of renewable resources as sustainable raw materials in the manufacture of UPRs [6,7]. To address this challenge, there are complex requirements to be fulfilled, especially when it comes to the choice of RD. A well-suited RD should have good compatibility with the prepolymer, ability to homopolymerize and copolymerize with unsaturation of the prepolymer under the given curing conditions, low volatility and most importantly, viscosity low enough to ensure good processability and properties of the final material.

Reports from the Pacific Northwest National Laboratory (PNNL) and the National Renewable Energy Laboratory (NREL) in the USA have identified 12 types of potential renewable building blocks [8]. Bozell et al. have further elaborated a top 10 list of bio-based products from biorefinery that were proposed by the Department of Energy, USA [9].

Novel monomers synthesized from renewable materials are extremely attractive. In this context, vinyl levulinate (VL), as one of the renewable RDs used for UPR synthesis, showed promising results with only 5.5 wt.% of the residual monomer inside the prepared network [10]. Still, the residual VL acts as a plasticizer and consequently, compared to styrene-based ones, VL-based UPRs exhibit a lower α relaxation (T_α_ = 180 and 100 °C, respectively), lower elastic moduli at the rubbery plateau (G′ = 10^8^ and 10^7^ Pa). Strain at break (up to 1.8 ± 0.2%) and Charpy impact strength (up to 2.7 ± 0.3 kJ m^−2^) are comparable independently of the RD chemical nature.

Fatty acid monomers (FAM) were found to be excellent alternatives to styrene due to their low cost and low volatility [11]. Depending on the composition, UPRs containing FAM-based RDs exhibit the flexural strength up to 61 MPa [5]. They originated from plant oils, which are composed of more than 99% triglyceride molecules, containing 10 or more different fatty acids, which range in the length, level of unsaturation, reactivity and functionality [12]. Still, their development has been restricted due to the moderately high viscosity and low glass transition temperature of the cured resins [13].

With potential to be produced from biomass, (methyl)acrylate monomers were recognized as promising RD candidates in the formulation of vinyl ester resins (VERs) and UPRs. Generally, they have highly reactive (methyl)acrylate C=C bonds and relatively low molecular weights.

Considering the group of VERs, several different bio-based RD methacrylate functions have been reported as positive styrene alternatives [14]. On the other hand, a lack of reports dealing with the substitution of styrene with bio-based methacrylates in UPR formulations indicates the serious complexity of this subject. The main reason arises from the peculiar reactivity of fumarate groups with other double bonds. Copolymerization of fumarate groups with styrene is well-favored [15]; however, the situation is much more delicate with other groups of bio-based RD.

For example, hemp fiber-reinforced UPR composites modified with butyl methacrylate (BMA) showed the improved flexibility and toughness compared to unmodified ones [16]. Composites obtained from BMA-modified UPRs had 27.4, 63.0, and 36.6% greater elongation at break (up to 10%), flexural strain (up to 3.5%), and impact strength (up to 22 kJ/m^2^), respectively.

In a novel family of UPs derived from 2,5-furandicarboxylic acid (FDCA) and itaconic acid, 2-hydroxyethylmethacrylate (HEMA) was copolymerized to improve its fluidity and crosslinking degree. The obtained results showed that the increase in the HEMA content led to the increase of both the gel content and Tg of UPE resins [17]. The study on synthesis and characterization of UPs based on FDCA has also been reported by Sousa et al., wherein HEMA was used as a reactive solvent instead of styrene [18]. The obtained UPRs showed adequate thermal and mechanical behavioral tendencies, similar to those of petrochemical ones, which were reflected in the high glass transition temperature (up to 104 °C) and good thermal stability (up to 230 °C).

Meht et al. have used HEMA, isobornyl methacrylate (IBOMA) and methyl methacrylate (MMA) as RDs for itaconic acid-based UPs [19]. The obtained results have been quite promising in terms of the properties of these styrene-free bio-based UPRs, which were found to be equivalent to those of commercial styrene-based ones. At last, these methacrylates could be used as styrene alternatives to reduce the resin viscosity and also improve the resin crosslinking density, and provide feasible coating properties. Still, segments of these monomers may bond into the 3D network and are easy to break down at lower temperature, resulting in less thermally stable thermosets compared to pure UPRs. The additional problems are closely related to the low boiling points of BMA, HEMA and IBOMA, which could cause highly volatile emissions during processing [16,17,18,19]. A comprehensive overview of the thermomechanical properties of other different bio-based UPRs formulations has been reported previously [5].

In our previous work, we determined that UPRs based on itaconic acid, succinic acid and propylene glycol where dimethyl itaconate (DMI) was used as a reactive diluent have shown good applicative properties [20,21]. However, the viscosity of the prepared resins was higher than that of commercial resins, which limited their applications. In order to overcome this issue, in this paper we investigated the effect of the presence of MMA besides DMI in the mixture used as a reactive diluent. MMA was chosen since it can be obtained from renewable sources (from citric or itaconic acid), it has much lower viscosity than DMI and the copolymerization rate of DMI and MMA is similar to the homopolymerization rate of DMI [22].

## 2. Materials and Methods

The itaconic acid, succinic acid, 1,2-propandiol, hydroquinone and toluene were supplied from Acros Organics. The catalyst, zinc acetate, was purchased from Sigma-Aldrich. The initiator, methyl ethyl ketone peroxide (MEKPO), and reactive diluents used for preparation of cured resins, dimethyl itaconate (DMI) and methyl methacrylate (MMA), were all supplied by Sigma Aldrich. All chemicals were used as received.

### 2.1. Synthesis

The reaction of melt polycondensation with diacids and 1,2-propandiol was used for the synthesis of the prepolymer. The mole ratio of total diacids to 1,2-propandiol was 1:1.05, while the amount of itaconic acid was equimolar to that of succinic acid in each reaction. Toluene (0.5 wt.% based on monomers) was used to increase the rate of water removal. Hydroquinone (150 ppm) was added as a free radical scavenger and the components were mixed. The reaction was carried out in a closed system equipped with a stirrer, Dean–Stark and thermometer, and under nitrogen atmosphere.

**Scheme 1:**
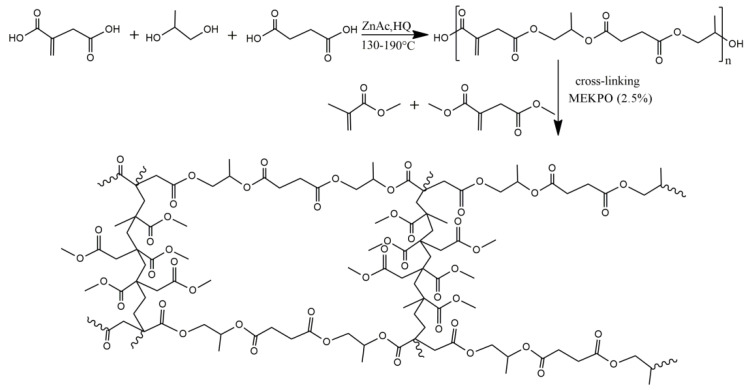
General mechanism of the polycondensation reaction and cross-linking.

The temperature range was 110–190 °C and was raised by 10 °C per hour. The reaction was carried out until the acid value reached 50. The chemical reaction is presented in Scheme 1.

The resin was cooled to 90 °C under nitrogen atmosphere and vacuumed. The so obtained prepolymer was divided into seven aliquots, subsequently dissolved in the reactive diluent (40% *w/w* with respect to the resin) containing both dimethyl itaconate (DMI) and methyl methacrylate in different weight ratios (100% DMI; 90% DMI, 10% MMA; 80% DMI, 20% MMA; 65% DMI, 35% MMA; 50% DMI, 50% MMA; 25% DMI, 75% MMA; 100% MMA).

The prepared UPRs were mixed with MEKPO (2.5% *w*/*w*), homogenized and poured into Teflon molds. The Teflon molds were placed in an air oven, left for 24 h at different temperatures (40 °C, 60 °C and 80 °C) to determine the optimal curing regime (completely cured samples with no cracks nor bubbles). Additionally, all the samples were kept at 120 °C for one hour to harden. The acid value (AV) was defined as the number of milligrams of KOH needed to neutralize 1 g of resin and was measured according to ASTM D465-01. Around 0.5 g of resin was titrated with a KOH solution in ethanol (0.1 mol/L).

### 2.2. Tensile Testing

The uniaxial tensile mechanical properties of the investigated UPR samples were evaluated using the Shimadzu Autograph AGS-X servo-hydraulic testing machine, equipped with a 1 kN load cell at ambient temperature according to ASTM D638. Five measurements were performed for each UPR sample at a testing rate of 0.5 mm min^−1^. The average values of the break stress and break stroke strain, the standard deviations and Young’s modulus were determined. Young’s modulus was calculated by the software TRAPEZIUM X from the linear part of the stress–strain curve.

### 2.3. Dynamic Mechanical Analysis

The dynamic mechanical properties of the UPR samples were analyzed by a Discovery HR-2 (TA Instruments, New Castle, DE, USA). The prism-shaped samples (60 × 12 × 2 mm) were exposed to the fixed strain amplitude of 0.1% and angular frequency of 1 Hz in the temperature range from 25 to 150 °C. The measured data were the storage modulus (G′/GPa), loss modulus (G″/MPa) and damping factor (tan δ), while the glass transition temperature (Tg) was determined as the temperature where tan δ showed the maximum value. All results were obtained from the second heating cycle.

### 2.4. Viscosity Measurements

Viscosities were measured isothermally at 25, 35, 45 and 55 °C using a Discovery HR-2 (TA Instruments, New Castle, DE, USA) in Peltier plate (±0.1 °C error) geometry. The sample (around 0.05 mL) was loaded in a 20 mm 1° steel cone with a truncation gap of 25 μm. The shear rate was increased stepwise from 0.1 to 100 s^−1^, collecting 21 data points to observe any non-Newtonian behavior. At the given shear rate, the shear stress was measured every 2 s. The data were recorded when the shear rate was stabilized with up to 5% tolerance for three consecutive points. UPRs were measured in triplicate and the viscosities were averaged and reported.

The gel content was calculated by extraction in tetrahydrofuran (THF). The cured samples were cut into rectangular shapes, with dimensions of 40 × 10 × 4 mm, and their weight was determined (*Wi*). Such prepared samples were immersed into THF for 28 days at room temperature. The insoluble fractions, which correspond to the inflated polymer network, were filtered, carefully dried under vacuum and then measured (*Wsol*). The soluble fractions (wt.%) were calculated as follows:

$$Wt.\left(\%\right)=\frac{Wsol}{Wi}\times 100$$

## 3. Results and Discussion

As already mentioned, bio-based UPR was prepared by mixing 60 wt.% of synthesized UP and 40 wt.% of DMI as a reactive diluent. In order to reduce viscosity of the UPR, a certain amount of dimethyl itaconate was replaced by the same amount of MMA, whereby the following DMI/MMA weight ratios were applied: 100% DMI; 90% DMI, 10% MMA; 80% DMI, 20% MMA; 65% DMI, 35% MMA; 50% DMI, 50% MMA; 25% DMI, 75% MMA; 100% MMA.

**Figure 1:**
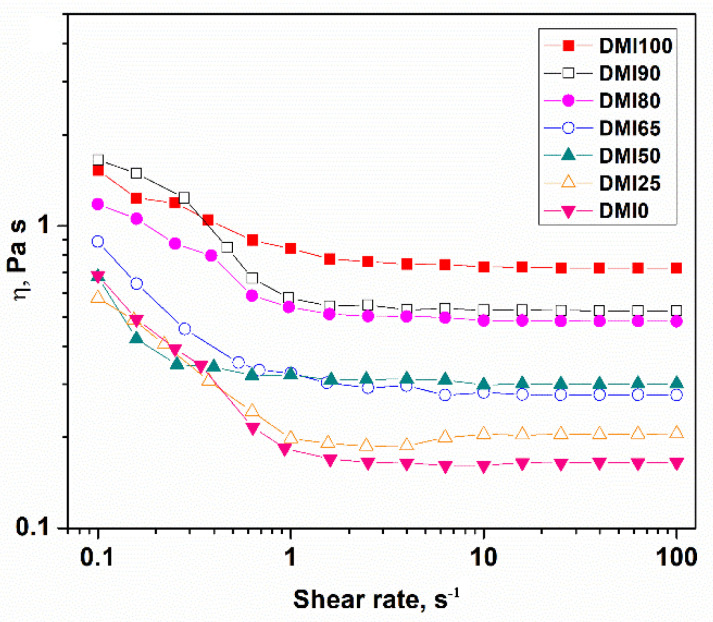
Viscosity as a function of the shear rate of the examined UP resins, measured at 25 °C.

Shear rate dependence of the viscosity of examined UPR samples measured at 25 °C is shown in Figure 1.

At shear rates higher than 1 s^−1^, the viscosity of the UPRs was found to be independent of the shear rate. This behavior is characteristic of Newtonian liquids and it indicates an absence of chain entanglements. The viscosity of the UPRs decreased with an increasing amount of MMA in the reactive diluent composition. This was a result of the much lower viscosity and lower polarity of MMA compared to DMI. These UPRs represented a concentrated solution of oligomeric UP molecules in the reactive diluent. The viscosity of UPRs is not only influenced by the interactions of UP molecules with reactive diluent molecules, but also by interactions of UP molecules with other UP molecules [20]. The molecules of the reactive diluent penetrate between the molecules of UP, separate them and decrease the intermolecular interactions between them, leading to the decrease of the viscosity of UPRs. In this case, less polar MMA molecules caused greater decrease of intermolecular interactions between the larger and polar UP molecules than the more polar DMI molecules.

**Figure 2:**
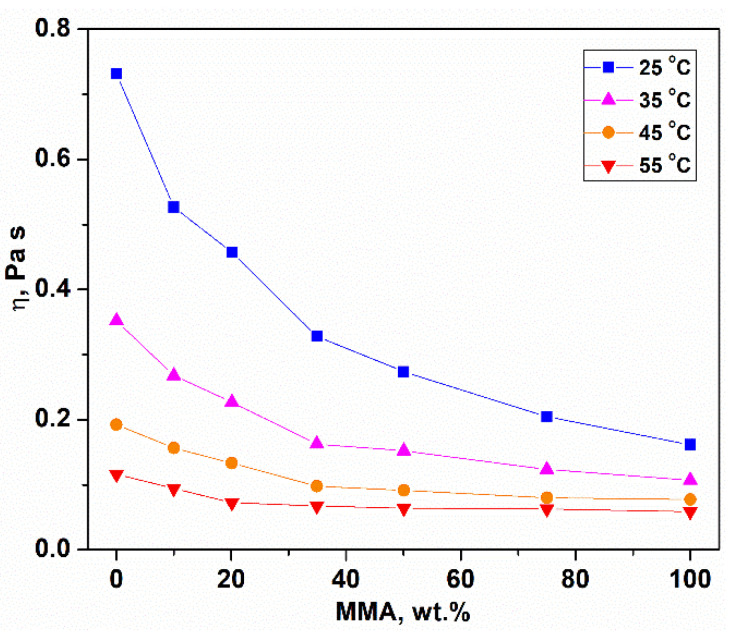
Viscosity as a function of temperature of the examined UP resins.

Therefore, the viscosity of the UPRs decreased with an increasing amount of MMA in the reactive diluent composition. The temperature’s influence on the viscosity of the UPRs was examined in the temperature range from 25 to 55 °C and obtained results are shown in Figure 2.

Figure 2 shows that the viscosity of the UPRs decreased with increasing temperature. It is known that UPRs with viscosity lower than 0.5 Pa s, at processing temperature, can be used for composite production [23]. Since all examined UPR samples had viscosity lower than 0.5 Pa s at 35 °C, they could be used for that purpose. According to this request, the UPR samples which contained at least 20 wt.% MMA could be used even at 25 °C. The measured viscosity was plotted against the shear rate and the data for shear rates up to 1 s^−1^ were fitted using the Ostwald–de Waele power law equation:

$$\eta \;=\;\kappa \gamma^{n-1}$$

where η is resin viscosity, γ is shear rate, κ is the magnitude of viscosity, with higher values indicating more viscous resins, and n the flow behavior index, where lower values indicate greater shear thinning. Obtained values of Ostwald–de Waele parameters are presented in Table 1. Increasing the DMI concentration increased the magnitude of viscosity (κ, Pa s). On the other hand, the lower viscosity resins showed some mild shear thinning behavior.

**Figure 3:**
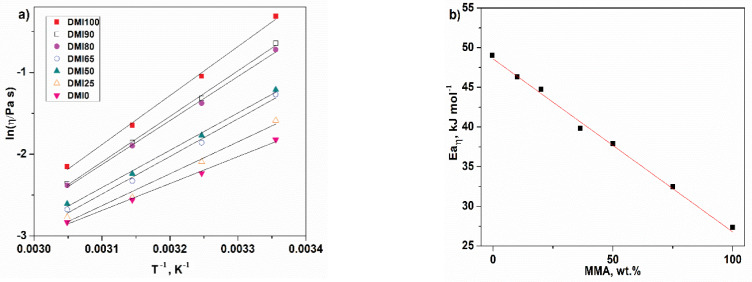
(**a**) ln η as a function of 1/T at the constant shear rate (10 s^−1^); (**b**) Ea as a function of MMA content in the reactive diluent.

The reciprocal temperature dependence of viscosity logarithm at a constant shear rate (10 s^−1^) for all examined UPR samples is shown in Figure 3a. A well-known method to correlate the viscosity of polymer solution with temperature is the Arrhenius equation:

η=η∞ eEaRT

where η_∞_ is infinite viscosity, Ea is activation energy of flow at the constant shear rate, R is the gas constant (8.314 J/mol K) and T is temperature in K.

Activation energy was calculated from the slope of the obtained linear dependence ln η vs. 1/T (Figure 3a) and obtained values are shown as a function of the MMA content in the reactive diluent in Figure 3b. As seen, the activation energy decreased linearly with an increasing MMA amount in the reactive diluent composition, indicating that the increase of both MMA and temperature reduces viscosity by disrupting intermolecular interactions [24].

The curing of UP is particularly important due to the wide use of these polymers in a large number of industrial processes and applications. Determination of the optimal curing parameters leads to the promotion of the processing method in terms of the time and cost reduction and improvement of the final properties. The cure reaction occurs via a free radical reaction mechanism and is quite complex. Complexity is a consequence of the presence of three co-monomers (DMI, MMA and prepolymer) and rapid changes in the system rheology. Namely, copolymerization leads to the early formation of microgels, even at conversion levels as low as 3–4% [25,26]. The rapid exothermic reaction leads to the increase in temperature while microgels formation omits a good heat transfer, which could lead to local overheating and the possibility of reactive diluent evaporation. This is especially evident when a highly volatile compound is used, such as MMA. The evaporation of the reactive diluent leads to the formation of small bubbles throughout the material, which greatly impairs the final material properties. Therefore, we firstly investigated curing of novel resins through varying the amount of the initiator, curing temperature and duration of the post-cure hardening at elevated temperature in order to determine the optimal curing parameters. The optimal curing parameters were determined by visual and microscopic observation of cured samples, as well as by determination of the gel content. The curing parameters were decided when samples showed no porosity and the highest values of gel content were chosen as optimal. These were found to be 2.5 wt.% of MEKPO, curing temperature of 60 °C for 24 h and one hour of post-cure hardening at 120 °C. Samples cured under these conditions showed no visible defects. The values of gel content of these samples, presented in Table 2, were above 95%, except for the sample cured with pure MMA, where gel content was 93.8%. This could be a result of a higher amount of MMA oligomers in this sample. Fernandez-Garcia and Madruga have investigated copolymerization of DMI and MMA and found that the DMI-MMA radical is more reactive toward the DMI monomer than toward the MMA monomer [27]. Considering the similarity of unsaturations in prepolymers and DMI, at the early stages of UPR curing, MMA radicals have a higher tendency to react with unsaturation from prepolymers than with the MMA monomers. Therefore, at the end of curing the prepolymer unsaturations are mostly depleted, which further means that there is a higher chance for the formation of MMA oligomers or small molecular weight polymers. These MMA oligomers and polymers are easily dissolved in THF, which leads to the lower gel content. The increase in the DMI content results in the increase in the gel content with the exception of the sample cured with pure DMI. This could be due to the significantly higher viscosity of the sample diluted by pure DMI and the fact that the polymerization propagation constant is found to be diffusion controlled [28,29].

**Figure 4:**
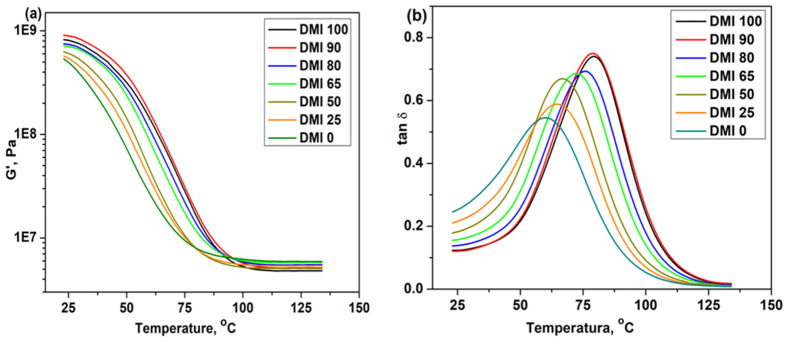
The dependence of storage modulus (**a**) and tan δ (**b**) on temperature in prepared samples.

The macroscopic properties of UPR depend on its microstructure, mainly on the average number of crosslinks between chains (crosslink density) and the average length of the segments between crosslinks. The crosslink density depends on the structure of the reactive compounds (prepolymer and reactive diluent(s)) and the ability of different types of unsaturations to copolymerize. The average length of the crosslinks depends not only on the relative amounts of prepolymer and reactive diluent(s) but also on the behavior of the radical copolymerization. For example, if one comonomer has higher tendency to homopolymerize, then the crosslink density will be lower, while the length of the segments between crosslinks will be larger. On the other hand, if the tendency toward copolymerization is more pronounced, then some unreacted monomers will remain at the end of curing. These monomers may act as plasticizers and thus, affect the global static mechanical properties, such as stiffness. Dynamic mechanical analysis was performed in order to perceive the network structure of cured samples. The storage modulus-temperature and tan δ-temperature curves are presented in Figure 4. The experimental crosslink density, *n_e_* (Table 2) was calculated based on the kinetic theory of rubber elasticity from the rubbery modulus using the following equation [30]:

$$G^{\prime }=3n_{e}RT,$$

where *G*′ is storage modus (GPa), *T* the temperature (K) and *R* the universal gas constant (8.3145 J/Kmol).

The lowest crosslink density was determined for the sample cured with pure DMI. This was a consequence of this sample having the highest viscosity, which impeded crosslinking. However, there was no clear correlation between the crosslink density and ratio of DMI to MMA, indicating that both monomers similarly reacted with unsaturated prepolymers. These results were expected considering good copolymerization between DMI and MMA [31] and a similar structure of prepolymer unsaturation and DMI. On the other hand, the increase in the DMI content in the reactive diluent led to the increase in the storage modulus. The G′ in the glass region is primarily dictated by both the strength of intermolecular forces existing between polymer chains and the arrangement of the polymer chains’ packing [32]. The increase in G′ with increasing DMI content could be a consequence of a stronger electrostatic interaction of two carbonyl groups in DMI compared to only one in MMA. The more pronounced electrostatic interaction led to the increase in the overall intermolecular interactions, which further resulted in the increase in G′. At higher temperatures these electrostatic interactions are broken and their effect is negligible. Furthermore, decrease in G′ values with increasing MMA content could be attributed to the presence of nanopores or micropores that weaken material mechanical properties. Namely, MMA is significantly more volatile than DMI. Thus, during early stages of crosslinking, when the temperature increases due to the exothermic crosslinking and pure heat dissipation, some of the MMA monomer boiled, creating nanopores or micropores throughout the material.

The tan δ-temperature curves of the UPR networks in the α-relaxation region are presented in Figure 4b. With increasing DMI content, the curves’ maxima, i.e., the apparent glass transition temperatures, systematically shifted to higher temperatures (Table 2). Furthermore, the shape of the curve (intensity and broadness) changed accordingly. Apparently, the DMI/MMA ratio affects the structure of the resulting networks. The intensity and broadness are sensitive to the amplitude and homogeneity of the macromolecular chain motions. The increase in the MMA content decreases the intensity and increases the broadness of the tan δ-temperature curve. This behavior indicates the rise in the heterogeneity of formed networks with increasing MMA. Considering the higher MMA reactivity and tendency to react with unsaturation in prepolymers, the observed heterogeneity increment could mainly be attributed to the formation of tighter microgel structures. This microstructure is composed of distinct regions of the densely crosslinked network distributed among a loosely crosslinked matrix. As mentioned before, DMI exhibits stronger electrostatic interaction compared to MMA. This more pronounced electrostatic interaction led to the increase in the overall intermolecular interactions, which further resulted in the increase in T_g_, shown as a shift of tan δ maximum towards higher temperatures.

**Figure 5:**
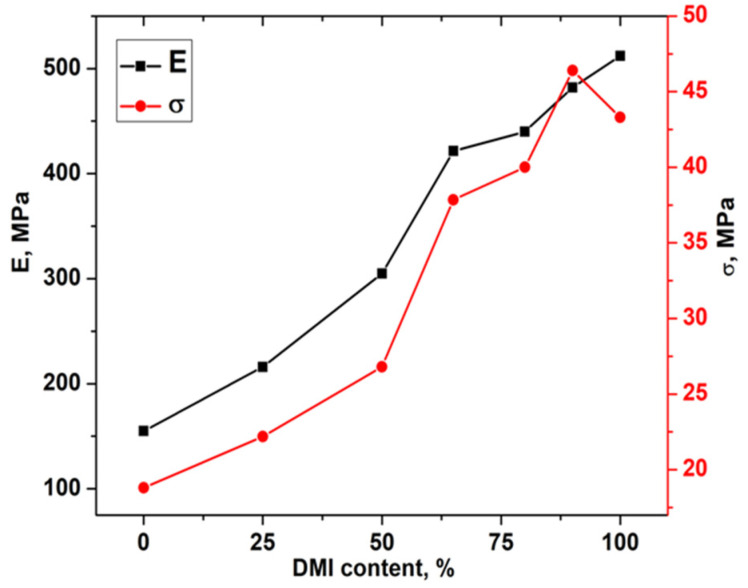
Tensile strength and Young’s modulus of the cured samples.

The mechanical properties of prepared resins were investigated by uniaxial tensile testing. Stress-strain curves of each sample were of a shape that corresponded to their brittle nature. The obtained results for the tensile strength and Young’s modulus are given in Figure 5. With increasing DMI content in the reactive diluent, both the tensile strength and elastic modulus increased. As already mentioned, the increase in DMI led to formation of a more homogenous network and stronger overall intermolecular interactions.

On the other hand, the increase in MMA content resulted in an increase in the porosity. It is well-known fact that defects in a material have more pronounced effects on the tensile than on dynamic-mechanical properties. This behavior was observed in our work. Namely, the highest storage modulus was 75.4% higher than the lowest storage modulus (G′_DMI90_/G′_DMI0_ = 0.754), whereas the highest σ value was 147% higher than the lowest σ value (σ _DMI90_/σ _DMI0_ = 2.47). The continuous increase in the tensile strength with increasing DMI content can be observed in Figure 5. Elastic modulus also increased with the increase in DMI content until the sample cured with pure DMI. This drop in the elastic modulus could be explained by the plasticizing effect of unreacted DMI: the gel content of the sample DMI 90 was 97.7%, whereas that of the sample DMI 100 was 95.4% (Table 1). It is a well-known fact that plasticizer affects more pronouncedly the modulus of elasticity than the tensile strength [33]. Thus, the elastic modulus of DMI 100 is lower than modulus of DMI 90, whereas the tensile strength of DMI 100 is higher than that of DMI 90. The sample toughness was determined from the area under the stress-strain curve and obtained results are given in Table 2. The increase in the DMI content caused an increase in the material toughness, except for in the sample cured with pure DMI.

## 4. Conclusions

Change toward a circular industry requires rethinking, redesigning and rewiring polymeric technologies. In this way, we aim to reduce the environmental footprint with responsible production and products. Currently, it is difficult to completely replace petroleum-based polymer materials with pure bioresource-constructed polymer materials due to deficiencies in some performances. Thus, it is more realistic to develop partially substituted petroleum polymer materials. In this work, we improved the rheological properties of fully bio-based unsaturated polyester resin by utilizing a mixture of bio-based and petroleum based reactive diluent, dimethyl itaconate and methyl methacrylate, respectively. The increase in the methyl methacrylate content caused a decrease in the resin viscosity due to the disruption of intermolecular interactions of prepolymer macromolecules. The viscosity was reduced from 732 to 162 mPa s by replacing dimethyl itaconate with methyl methacrylate, which greatly enhanced the applicative potential. On the other hand, the addition of methyl methacrylate led to the decrease of the mechanical and dynamic-mechanical properties by 147% and 75%, respectively. The glass transition temperature of the samples cured by pure dimethyl itaconate was 79.3 °C and decreased to 60.4 °C when dimethyl itaconate was replaced with pure methyl methacrylate in the curing process. However, the substitution of a small amount (10 wt.%) of dimethyl itaconate with methyl methacrylate caused a decrease in the resin viscosity of 30%, and a slight improvement of the mechanical and dynamic-mechanical properties. Thus, this work suggests that commercially viable unsaturated polyester resins, with a high amount of renewable carbon, could be prepared by utilizing a mixture of nine parts of dimethyl itaconate and one part of methyl methacrylate as a reactive diluent. The low viscosity and good mechanical properties of these resins facilitates potential usage for the production of various composite materials. Low viscosity is an especially important factor because it enables resins to be processed by higher number of techniques such as resin transfer molding, vacuum infusion processing, filament wilding, centrifugal casting and continuous lamination.

## Figures and Tables

**Table 1:** Ostwald–de Waele parameters and viscosity of the resins at 25 °C.

Sample	DMI 100	DMI 90	DMI 80	DMI 65	DMI 50	DMI 25	DMI 0
η, mPa s	732	527	457	328	274	205	162
κ, Pa s	0.800	0.585	0.501	0.321	0.292	0.201	0.173
n	0.727	0.618	0.614	0.584	0.605	0.458	0.398

**Table 2:** The UPR storage modulus at 25 °C (*G′*) and 130 °C (*G′*_130°C_), crosslink density (*n_e_*), *T_g_*, gel content and toughness.

Sample	*G′,* GPa	*G′*_130°C_, MPa	*n_e_*, mol m^−3^	*T_g_*, °C	Gel Content, %	Toughness, J m^−3^
DMI 100	0.81	4.82	486	79.3	95.4	0.270
DMI 90	0.86	5.14	518	78.8	97.7	0.410
DMI 80	0.74	5.52	556	76.1	97.9	0.410
DMI 65	0.71	5.75	579	72.1	97.0	0.298
DMI 50	0.60	5.06	510	66.8	95.9	0.280
DMI 25	0.54	5.40	544	64.7	95.3	0.242
DMI 0	0.49	5.92	597	60.4	93.8	0.210

## Data Availability

Data presented in this study are available on request from the corresponding author.
